# Differentiation Between Primary Central Nervous System Lymphoma and Atypical Glioblastoma Based on MRI Morphological Feature and Signal Intensity Ratio: A Retrospective Multicenter Study

**DOI:** 10.3389/fonc.2022.811197

**Published:** 2022-01-31

**Authors:** Yu Han, Zi-Jun Wang, Wen-Hua Li, Yang Yang, Jian Zhang, Xi-Biao Yang, Lin Zuo, Gang Xiao, Sheng-Zhong Wang, Lin-Feng Yan, Guang-Bin Cui

**Affiliations:** ^1^Department of Radiology and Functional and Molecular Imaging Key Lab of Shaanxi Province, Tangdu Hospital, Fourth Military Medical University, Xi’an, China; ^2^Battalion of the First Regiment of cadets of Basic Medicine, Fourth Military Medical University, Xi’an, China; ^3^Battalion of the Second Regiment of cadets of Basic Medicine, Fourth Military Medical University, Xi’an, China; ^4^Department of Radiology, Xi’an XD Group Hospital, Shaanxi University of Chinese Medicine, Xi’an, China; ^5^Department of Radiology, West China Hospital, Sichuan University, Chengdu, China

**Keywords:** primary central nervous system lymphoma, glioblastoma, magnetic resonance imaging, signal intensity ratio, morphological feature

## Abstract

**Objectives:**

To investigate the value of morphological feature and signal intensity ratio (SIR) derived from conventional magnetic resonance imaging (MRI) in distinguishing primary central nervous system lymphoma (PCNSL) from atypical glioblastoma (aGBM).

**Methods:**

Pathology-confirmed PCNSLs (n = 93) or aGBMs (n = 48) from three institutions were retrospectively enrolled and divided into training cohort (n = 98) and test cohort (n = 43). Morphological features and SIRs were compared between PCNSL and aGBM. Using linear discriminant analysis, multiple models were constructed with SIRs and morphological features alone or jointly, and the diagnostic performances were evaluated *via* receiver operating characteristic (ROC) analysis. Areas under the curves (AUCs) and accuracies (ACCs) of the models were compared with the radiologists’ assessment.

**Results:**

Incision sign, T_2_ pseudonecrosis sign, reef sign and peritumoral leukomalacia sign were associated with PCNSL (training and overall cohorts, *P* < 0.05). Increased T_1_ ratio, decreased T_2_ ratio and T_2_/T_1_ ratio were predictive of PCNSL (all *P* < 0.05). ROC analysis showed that combination of morphological features and SIRs achieved the best diagnostic performance for differentiation of PCNSL and aGBM with AUC/ACC of 0.899/0.929 for the training cohort, AUC/ACC of 0.794/0.837 for the test cohort and AUC/ACC of 0.869/0.901 for the overall cohort, respectively. Based on the overall cohort, two radiologists could distinguish PCNSL from aGBM with AUC/ACC of 0.732/0.724 for radiologist A and AUC/ACC of 0.811/0.829 for radiologist B.

**Conclusion:**

MRI morphological features can help differentiate PCNSL from aGBM. When combined with SIRs, the diagnostic performance was better than that of radiologists’ assessment.

## Introduction

Preoperative distinguishing primary central nervous system lymphoma (PCNSL) from glioblastoma (GBM) is of highly clinical relevance because treatment strategies for the two diseases vary substantially. In patients with GBM, surgical resection followed by concurrent chemoradiation is the first-line treatment, whereas patients with PCNSL usually undergo stereotactic biopsy followed by high-dose methotrexate (1, 2). Moreover, preoperative application of steroids may affect the histopathologic diagnosis of PCNSL (2). Therefore, reliable preoperative differentiation of both entities is important.

Conventional magnetic resonance (MR) imaging features allow distinguishing PCNSL from typical GBM for most patients because PCNSL in an immunocompetent patient usually manifests as a homogeneously enhanced mass lesion on contrast-enhanced T_1_-weighted (T_1_CE) images. And typical GBM usually exhibits an irregular rim-like enhancement with necrosis (3, 4). However, this enhancement pattern is not reliable in cases of atypical glioblastoma (aGBM) with no visible necrosis, which complicates the discrimination between aGBM and PCNSL (5, 6).

Both conventional and advanced MR techniques have been reported to be helpful in differentiating PCNSL from GBM (7–12). However, most of these studies enrolled all GBM patients, which can be differentiated from PCNSL based on findings of conventional MRI in most cases. A few studies on differentiating PCNSL from aGBM involve advanced imaging sequences or radiomics strategy (5, 6, 13, 14). Despite great advances, these techniques are associated with increased costs and postprocessing time and may not be routinely adopted by every patient in clinical practice. In contrast, T_2_-weighted imaging (T_2_WI), T_1_-weighted imaging (T_1_WI), and T_1_CE imaging are almost always available. Systematic evaluation of MRI morphological features of PCNSL and aGBM is, however, lacking. As an important supplement to subjective analysis, easily obtained quantitative parameters can further provide diagnostic information. Considering the pathophysiological difference between PCNSL and aGBM may be reflected in the form of signal intensity ratio (SIR), whether SIR analysis is effective in distinguishing aGBM from PCNSL remains largely unknown.

Here, we endeavored to compare morphological features and analyze SIR based on conventional MR sequences (T_1_WI, T_2_WI, and T_1_CE) to develop a quick and easy tool for differentiation of PCNSL and aGBM.

## Materials and Methods

Ethics review board approvals from three institutions were obtained, and written informed consent was waived for this retrospective study.

### Patients

Potentially eligible patients from Tangdu Hospital (from January 2012 to June 2021), XD Group Hospital (from January 2015 to May 2021), and West China Hospital (from January 2016 to June 2021) were identified with pathologically proven PCNSL or GBM.

Inclusion criteria were as follows: 1) no prior treatment history before MR examination, including biopsy, surgery, radiotherapy, chemotherapy, or corticosteroid treatment; 2) pretreatment MRI with conventional sequences available, including axial T_1_WI, T_2_WI, and T_1_CE imaging; 3) no hemorrhage inside the tumor based on T_1_WI and T_2_WI; 4) all PCNSL patients were immunocompetent. The exclusion criteria were as follows: 1) typical GBM with visible necrosis; 2) poor image quality with motion artifacts or susceptibility; 3) intracranial metastasis from systemic lymphoma. Atypical GBM was defined as solid enhancement with no visible necrosis based on axial T_2_WI and T_1_CE imaging, which were evaluated by two independent raters (YY and GX, with 5 and 10 years of experience in neuro-oncology imaging, respectively). When discrepancy exists, consensus was reached through discussion with a senior radiologist (G-BC, with 27 years of experience in brain tumor diagnosis).

**Figure 1:**
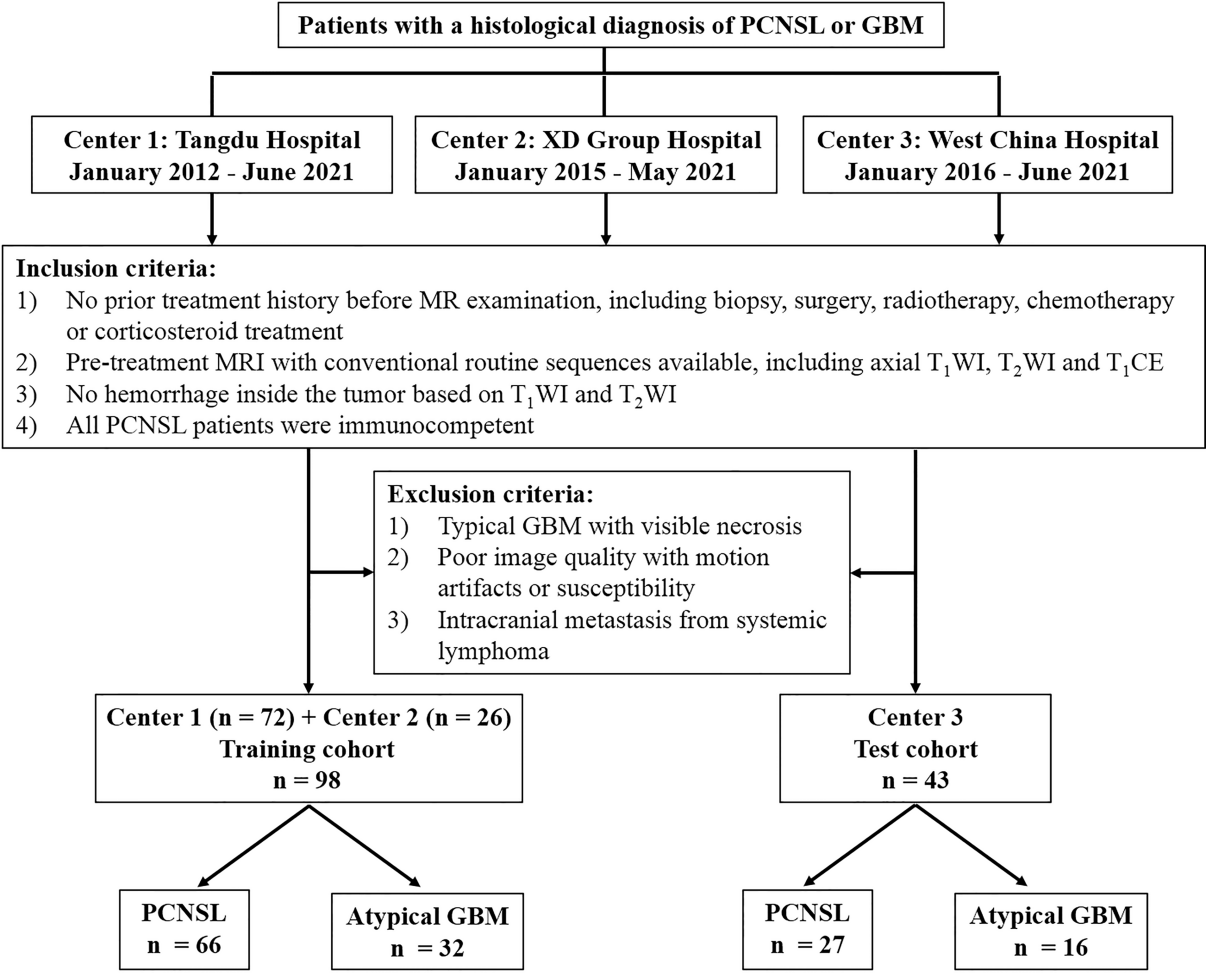
Flow diagram for patient selection.

According to the inclusion and exclusion criteria, 98 patients (center 1, n = 72; center 2, n = 26) with pathologically proven PCNSL (n = 66) or aGBM (n = 32) were consecutively enrolled and comprised the training cohort. Another cohort of 43 patients from center 3 with a diagnosis of PCNSL (n = 27) or aGBM (n = 16) comprised the external test cohort. The flow diagram for patient selection is shown in **Figure 1**.

### MR Image Acquisition

MRI scans were performed at three institutions with different protocols and various scanners. The routine sequences included axial T_1_WI, T_2_WI, and T_1_CE imaging. The detailed MRI parameters are provided in **Table S1** in the **Supplementary Material**. All patient names were de-identified prior to analysis.

### Image Analysis

Qualitative morphological features, which were characterized based on the criteria outlined in **Table 1**, were analyzed independently by two neuroradiologists (YY and GX), who were blinded to the final results. The inconsistency between them was resolved by discussion with a third senior neuroradiologist (G-BC). Notably, reef sign, peritumoral leukomalacia sign, and T_2_ pseudonecrosis sign were defined in our study for the first time (representative cases, see **Supplementary Material Figure S1**).

**Table 1:** MRI morphological feature definition.

Variable	Classification criteria
Localization	
Only supratentorial	The location of the tumor is supratentorial
Only infratentorial	The location of the tumor is infratentorial
Supra- and infratentorial	The location of the tumor is both infratentorial and supratentorial
Lesion type	
Solitary demarcated	Solitary tumor with demarcated boundary
Multiple demarcated	Multiple tumor with demarcated boundary
Solitary infiltrative	Solitary tumor with infiltrative boundary
Multiple infiltrative	Multiple tumor with infiltrative boundary
Streak-like edema	The shape of peritumoral edema is streak-like
Incision sign	Based on the T_1_CE images (axial, sagittal, or coronal plane), there are 1–2 umbilical concave or striated defects on the edge of the enhanced lesion
Reef sign	Single or multiple reef-like foci present as hypointensity on T_1_WI, hyperintensity on T_2_WI, and brighter signal within contrast-enhanced area of the lesion
Butterfly sign	Lesion involving the corpus callosum can infiltrate transcallosally, appearing as a symmetric “butterfly” appearance on T_1_CE imaging
Angular sign	The irregular enhancement lesions protrude to a certain direction, showing a sharp angle appearance
Peritumoral leukomalacia sign	The area adjacent to the tumor shows hypointensity on T_1_WI, hyperintesity on T_2_WI, and no contrast enhancement on T_1_CE imaging
T2 pseudonecrosis sign	On T_2_WI, the edge of the tumor is isointense to slightly hyperintense (gray matter as reference), accompanied by hyperintensity within the tumor. After the injection of contrast agent, the entire tumor shows significant and uniform enhancement
Involvement of structures	
Central structures	Involvement of basal ganglia, thalamus, or brainstem
Cortex	Involvement of cortex
Subventricular zone	Involvement of subventricular zone
Corpus callosum	Involvement of corpus callosum

ITK-SNAP software (version 3.8.0; http://itksnap.org) was used for SIR analysis (15). The abovementioned two neuroradiologists independently placed region of interest (ROI) for further consistency testing. The details of ROI placement strategy are shown in **Figure S2** and **Table S2** in **Supplementary Material**. Finally, four quantitative parameters, including T_2_ ratio (rT_2_), T_1_ ratio (rT_1_), T_1_CE ratio (rT_1_CE), and rT_2_/rT_1_ ratio (T_2_/T_1_), were obtained for each patient. The calculation formula is as follows:

rT_2_ = mean signal intensity of the lesion (SI_lesion_) on T_2_WI mean signal intensity of contralateral normal white matter (SI_control_)

rT_1_ = SI_lesion_ on T_1_WI/SI_control_

rT_1_CE = SI_lesion_ on T_1_CE/SI_control_

T_2_/T_1_ = rT_2_/rT_1_

### Radiologist’s Assessment

Two neuroradiologists (LZ and L-FY, with 10 and 17 years’ experience in radiology, respectively) independently reviewed the images. All radiologists had no prior knowledge of exact number of each entity and the final results. They can only have access to conventional MR images (T_1_WI, T_2_WI, and T_1_CE). Diagnosis was based on subjective analysis according to their clinical experience. The final diagnosis was recorded using a 4-point scale (1 = definite GBM; 2 = likely GBM; 3 = likely PCNSL; and 4 = definite PCNSL). To assess intra-observer agreement, radiologists reevaluated images after a 2-month washout period.

### Statistical Analysis

All statistical analyses were performed with SPSS 20.0 software (IBM Corp., Chicago, IL, USA) and R software version 3.6.1 (http://www.R-project.org). The normal distribution of data was investigated with Kolmogorov–Smirnov test. Numerical variables with normal distribution were denoted as mean and standard deviation. Continuous and categorical variables were compared using two-sample *t*-test and Fisher’s exact test, respectively. The intraclass correlation coefficient (ICC) was used to test the consistency of SIRs between the two radiologists. Intra-observer agreements of radiologist’s assessment were evaluated with Cohen’s kappa coefficient. Linear discrimination analysis (LDA) models for distinguishing aGBM from PCNSL were constructed with SIRs and morphological features alone or jointly. Receiver operating characteristic (ROC) analysis was performed to determine the performance of radiologists’ assessment and different models in the training, test, and overall cohorts, and accuracy (ACC) and area under the curve (AUC) were obtained. *P* < 0.05 indicated a significant difference.

## Results

### Demographic Characteristics

Patient demographic characteristics are summarized in **Table 2**. In this study, 93 PCNSLs (47 men, 46 women; mean age, 58.49 ± 12.56 years) and 48 aGBMs (29 men, 19 women; mean age, 55.12 ± 10.9 years) were enrolled. There were no significant differences in age and gender distribution between the two diseases (all *P* > 0.05). The vast majority of patients (83 out of 93 PCNSLs and 47 of 48 aGBMs) received surgical resection. Patients in the PCNSL group were pathologically confirmed as diffuse large B-cell lymphoma. Despite the diversity of clinical symptoms, headache, dizziness, or nausea was the most common initial symptom for patients with aGBM (44.1%, 41 out of 93) or PCNSL (41.7%, 20 out of 48).

**Table 2:** Baseline demographics of patients.

Variable	Training cohort (n = 98)			Test cohort (n = 43)			Overall cohort (n = 141)		
	PCNSL (n = 66)	aGBM (n = 32)	*P*	PCNSL (n = 27)	aGBM (n = 16)	*P*	PCNSL (n = 93)	aGBM (n = 48)	*P*
Age (mean ± SD)	60.3 ± 11.15	57.4 ± 9.84	0.236	55.16 ± 8.21	53.16 ± 8.21	0.528	58.49 ± 12.56	55.12 ± 10.9	0.119
Gender (N/%)			1.000			0.116			0.289
Male	54.5% (36/66)	56.3% (18/32)		40.7% (11/27)	43.8% (11/16)		50.5% (47/93)	60.4% (29/48)	
Female	45.5% (30/66)	43.7% (14/32)		59.3% (16/27)	56.2% (5/16)		49.5% (46/93)	39.6% (19/48)	
Symptoms (N/%)			NA			NA			NA
Headache/dizziness/nausea	48.5% (32/66)	34.4% (11/32)		33.3% (9/27)	56.2% (9/16)		44.1% (41/93)	41.7% (20/48)	
Visual disturbances	4.5% (3/66)	3.1% (1/32)		0	6.3% (1/16)		3.2% (3/93)	4.2% (2/48)	
Seizure	12.1% (8/66)	18.8% (6/32)		11.1% (3/27)	18.7% (3/16)		11.8% (11/93)	18.7% (9/48)	
Dysesthesia or hypesthesia	10.6% (7/66)	12.5% (4/32)		29.7% (8/27)	12.5% (2/16)		16.1% (15/93)	12.5% (6/48)	
Paresis	6.1% (4/66)	21.8% (7/32)		14.8% (4/27)	0		8.6% (8/93)	14.6% (7/48)	
Phatic disorder	9.1% (6/66)	0		0	0		6.5% (6/93)	0	
Psychiatric symptoms	9.1% (6/66)	9.4% (3/32)		11.1% (3/27)	6.3% (1/16)		9.7% (9/93)	8.3% (4/48)	
Pathologic procedure			NA			NA			NA
Biopsy	87.9% (58/66)	96.9% (31/32)		92.6% (25/27)	100% (16/16)		89.2% (83/93)	97.9% (47/48)	
Resection	12.1% (8/66)	3.1% (1/32)		7.4% (2/27)	0		10.8% (10/93)	2.1% (1/48)	

PCNSL, primary central nervous system lymphoma; aGBM, atypical glioblastoma. NA, not available.

### Comparison of MRI Morphological Features Between Primary Central Nervous System Lymphoma and Atypical Glioblastoma

MRI morphological features for both groups are shown in **Table 3**. Incision sign, reef sign, T_2_ pseudonecrosis sign, and peritumoral leukomalacia sign were detected in the PCNSL group but none in the aGBM group. Among them, reef sign and peritumoral leukomalacia sign were statistically different in both training (all *P* < 0.001) and test cohorts (reef sign, *P* = 0.003; peritumoral leukomalacia sign, *P* = 0.018). Similarly, significant statistical differences between the two groups were observed in incision sign and T_2_ pseudonecrosis sign based on the training cohort (all *P* < 0.001), whereas the differences in the test cohort were not statistically significant. Accounting for the small sample size of the test cohort, in order to increase the statistical power, we combined the training and test cohorts and performed statistical analysis on the overall cohort again. The results showed that incision sign and T_2_ pseudonecrosis sign were significantly different between the two groups (all *P* < 0.001). In addition, PCNSL was more likely to involve both supratentorial and infratentorial compartment than aGBM based on the overall cohort (*P* = 0.036). There were no significant differences in lesion type, streak-like edema, butterfly sign, angular sign, and involvement of structures between the PCNSL and aGBM groups (all *P* > 0.05).

**Table 3:** MRI morphological features in PCNSL and aGBM.

Variable	Training cohort (n = 98)			Test cohort (n = 43)			Overall cohort (n=141)		
	PCNSL (n = 66)	aGBM (n = 32)	*P*	PCNSL (n = 27)	aGBM (n = 16)	*P*	PCNSL (n = 93)	aGBM (n = 48)	*P*
Localization (N/%)			0.090			0.716			**0.036**
Only supratentorial	94% (62/66)	90.6% (29/32)		92.6% (25/27)	87.5% (14/16)		93.5% (87/93)	89.6% (43/48)	
Only infratentorial	1.5% (1/66)	9.4% (3/32)		3.7% (1/27)	12.5% (2/16)		2.2% (2/93)	10.4% (5/48)	
Supra- and infratentorial	4.5% (3/66)	0		3.7% (1/27)	0		4.3% (4/93)	0	
Lesion type (N/%)			0.180			0.111			0.974
Solitary demarcated	30.3% (20/66)	46.9% (15/32)		66.7% (18/27)	37.5% (6/16)		40.9% (38/93)	43.8% (21/48)	
Multiple demarcated	0	0		0	0		0	0	
Solitary infiltrative	37.9% (25/66)	21.9% (7/32)		33.3% (9/27)	62.5% (10/16)		36.6% (34/93)	35.4% (17/48)	
Multiple infiltrative	31.8% (21/66)	31.2% (10/32)		0	0		22.5% (21/93)	20.8% (10/48)	
Streak-like edema (N/%)			0.391			1.000			0.470
Yes	43.9% (29/66)	34.4% (11/32)		29.6% (8/27)	31.2% (5/16)		39.8% (37/93)	33.3% (16/48)	
No	56.1% (37/66)	65.4% (21/32)		70.4% (19/27)	68.8% (11/16)		60.2% (56/93)	66.7% (32/48)	
Incision sign (N/%)			**0.008**			0.069			**<0.001**
Yes	19.7% (13/66)	0		22.2% (6/27)	0		20.4% (19/93)	0	
No	80.3% (53/66)	100% (32/32)		77.8% (21/27)	100% (16/16)		79.6% (74/93)	100% (48/48)	
Reef sign (N/%)			**<0.001**			**0.003**			**<0.001**
Yes	42.4% (28/66)	0		40.7% (11/27)	0		41.9% (39/93)	0	
No	57.6% (38/66)	100% (32/32)		59.3% (16/27)	100% (16/16)		58.1% (54/93)	100% (48/48)	
Butterfly sign (N/%)			0.353			0.520			1.000
Yes	10.6% (7/66)	15.6% (5/32)		14.8% (4/27)	6.3% (1/16)		11.8% (11/93)	12.5% (6/48)	
No	89.4% (59/66)	84.4% (27/32)		85.2% (23/27)	93.7% (15/16)		88.2% (82/93)	87.5% (42/48)	
Angular sign (N/%)			0.556			0.386			1.000
Yes	13.6% (9/66)	18.7% (6/32)		18.5% (5/27)	6.3% (1/16)		15.1% (14/93)	14.6% (7/48)	
No	86.4% (57/66)	81.3% (26/32)		81.5% (22/27)	93.7% (15/16)		84.9% (79/93)	85.4% (41/48)	
Peritumoral leukomalacia sign (N/%)			**<0.001**			**0.018**			**<0.001**
Yes	30.3% (20/66)	0		29.6% (8/27)	0		30.1% (28/93)	0	
No	69.7% (46/66)	100% (32/32)		70.4% (19/27)	100% (16/16)		68.9% (65/93)	100% (48/48)	
T2 pseudonecrosis sign (N/%)			**0.004**			0.279			**< 0.001**
Yes	21.2% (14/66)	0		14.8% (4/27)	0		19.4% (18/93)	0	
No	78.8% (52/66)	100% (32/32)		85.2% (23/27)	100% (16/16)		80.6% (75/93)	100% (48/48)	
Involvement of structures (N/%)			0.072			0.704			0.121
Central structures	27.3% (18/66)	9.4% (3/32)		29.6% (8/27)	31.3% (5/16)		27.9% (26/93)	16.7% (8/48)	
Cortex	10.6% (7/66)	15.6% (5/32)		14.8% (4/27)	31.3% (5/16)		11.8% (11/93)	20.8% (10/48)	
Subventricular zone	9.1% (6/66)	18.8% (6/32)		11.1% (3/27)	6.25% (1/16)		9.7% (9/93)	14.6% (7/48)	
Corpus callosum	22.7% (15/66)	9.4% (3/32)		25.9% (7/27)	18.8% (3/16)		23.7% (22/93)	12.5% (6/48)	

The bold P value suggests a significant difference between the variables in the two cohorts.

PCNSL, primary central nervous system lymphoma; aGBM, atypical glioblastoma.

### Comparison of Signal Intensity Ratios Between Primary Central Nervous System Lymphoma and Atypical Glioblastoma

**Figure 2:**
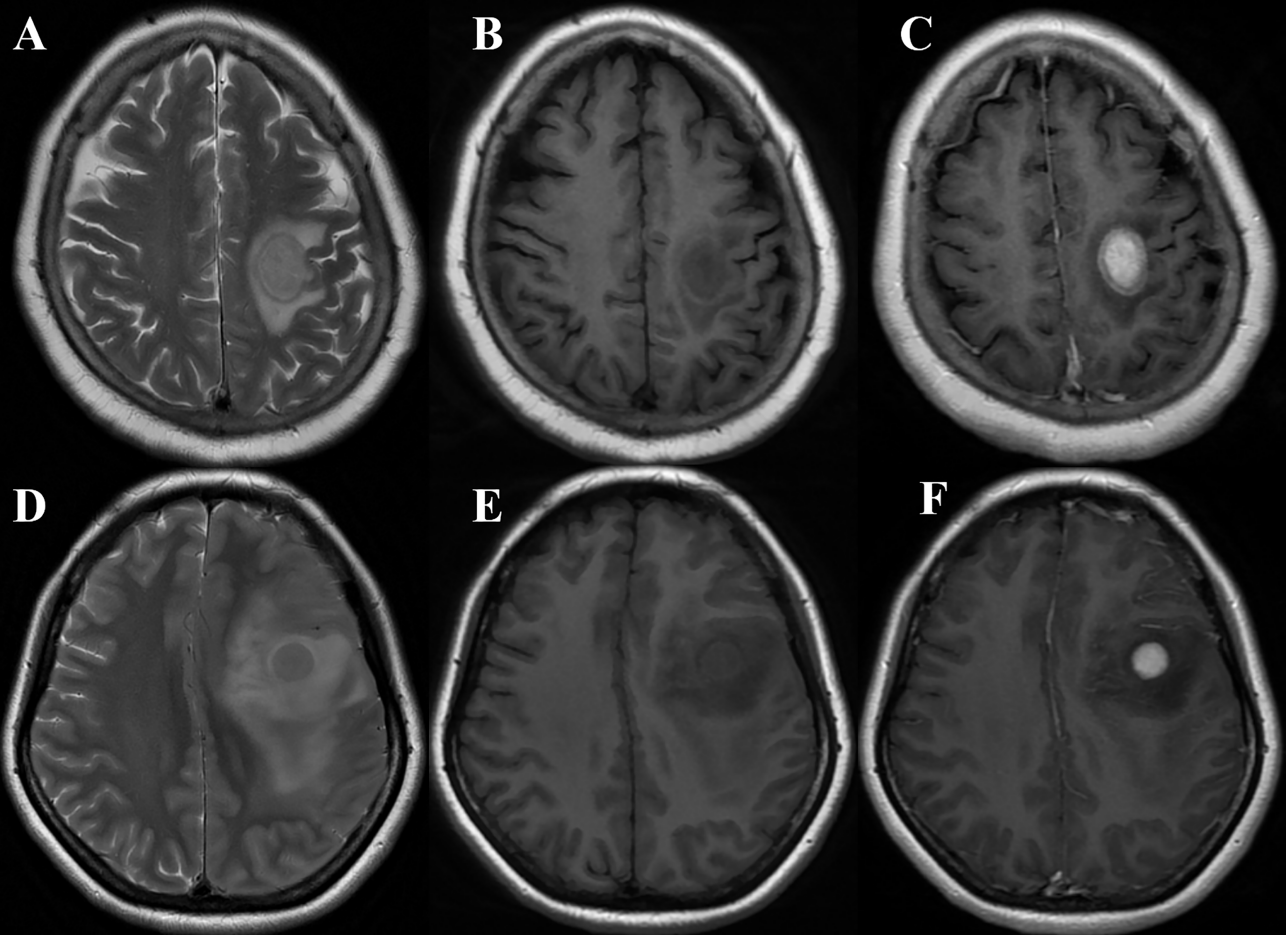
**(A–C)** A 68-year-old woman with primary central nervous system lymphoma (PCNSL) presented with left hemiparesis for 1 month. MRI showed a left frontal lobe lesion with iso- to slight hyperintensity on T_2_WI **(A)**, slight hypointensity on T_1_WI (**B**), and marked homogeneous enhancement on T_1_CE imaging **(C)** (take gray matter for reference). The quantitative parameters showed that rT1, rT2, T2/T1, and rT1CE were 0.65, 1.20, 1.82, and 1.87, respectively. The case was correctly diagnosed as PCNSL by models 1, 2, 4, and 5 and radiologist B while wrongly classified as glioblastoma (GBM) by radiologist A. **(D–F)** A 43-year-old woman with GBM presented with seizure. MRI showed a left frontal lobe lesion with isointensity on T_2_WI **(D)**, slight hypointensity on T_1_WI **(E)**, and marked homogeneous enhancement on T_1_CE imaging **(F)** (take gray matter for reference). The quantitative parameters showed that rT_1_, rT_2_, T_2_/T_1_, and rT1CE were 0.66, 1.46, 2.25 and 2.11, respectively. The case was correctly diagnosed as GBM by models 1, 2, 4, 5, and 6 and radiologist B while wrongly classified as PCNSL by radiologist A.

**Figure 3:**
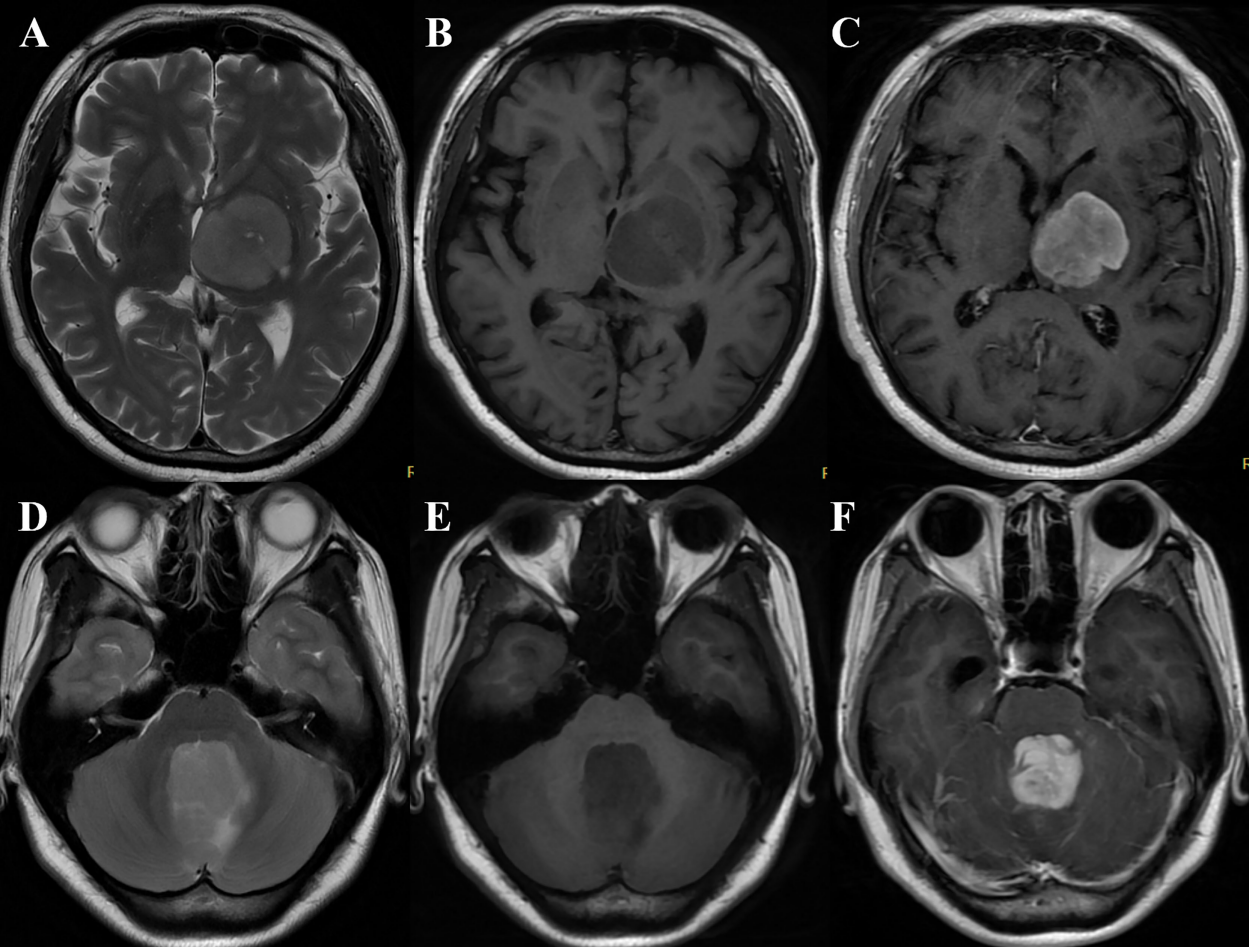
**(A–C)** A 60-year-old woman with primary central nervous system lymphoma (PCNSL) presented with right hemiparesis for 3 months. MRI demonstrated the lesion was located in the left basal ganglia and thalamus with slight hyperintensity on T_2_WI **(A)**, hypointensity on T_1_WI **(B)**, and marked heterogeneous enhancement on T_1_CE imaging **(C)** (take gray matter for reference). The quantitative parameters showed that rT_1_, rT_2_, T_2_/T_1_, and rT_1_CE were 0.83, 1.34, 1.62, and 1.23, respectively. The case was correctly diagnosed as PCNSL by models 1, 2, 4, 5, and 6 and radiologist B while wrongly classified as glioblastoma (GBM) by radiologist **(A)**. **(D–F)** A 23-year-old woman with GBM presented with nausea and vomiting for 2 months. MRI showed a vermis lesion with slight hyperintensity on T2WI **(D)**, hypointensity on T1WI **(E)**, and obvious homogeneous enhancement on T1CE imaging **(F)** (take gray matter for reference). The quantitative parameters showed that rT_1_, rT_2_, T_2_/T_1_, and rT_1_CE were 0.57, 1.91, 3.35, and 2.73, respectively. The case was correctly diagnosed as GBM by models 1, 2, 4, and 5 while wrongly classified as PCNSL by the two radiologists.

The rT_2_, rT_1_, T_2_/T_1_, and rT_1_CE values calculated for PCNSLs and aGBMs are summarized in **Table 4**. T_2_/T_1_ and rT_2_ values in aGBMs were significantly higher than those in PCNSLs in both the training and test cohorts (all *P* < 0.001). The rT_1_ value in aGBMs was significantly lower than that in PCNSLs (training cohort, *P* < 0.001; test cohort, *P* = 0.048). The rT_1_CE value of PCNSLs was slightly higher than that of aGBMs, but the difference was not statistically significant (all *P* > 0.05). The representative cases are shown in **Figures 2**, **3**.

**Table 4:** Quantitative MR signal intensity ratio comparisons between PCNSL and aGBM.

Variable	Training cohort (n = 98)			Test cohort (n = 43)			Overall cohort (n = 141)		
	PCNSL (n = 66)	aGBM (n = 32)	*P*	PCNSL (n = 27)	aGBM (n = 16)	*P*	PCNSL (n = 93)	aGBM (n = 48)	*P*
rT_2_	1.259 ± 0.113	1.690 ± 0.364	**<0.001**	1.297 ± 0.139	1.645 ± 0.239	**<0.001**	1.269 ± 0.121	1.675 ± 0.326	**<0.001**
rT_1_	0.629 ± 0.176	0.464 ± 0.118	**<0.001**	0.658 ± 0.131	0.570 ± 0.138	**0.048**	0.638 ± 0.164	0.499 ± 0.134	**<0.001**
T_2_/T_1_	2.159 ± 0.625	3.839 ± 1.163	**<0.001**	2.028 ± 0.384	3.049 ± 0.851	**<0.001**	2.121 ± 0.567	3.576 ± 1.125	**<0.001**
rT_1_CE	2.431 ± 0.564	2.198 ± 0.475	0.532	2.295 ± 0.489	2.065 ± 0.724	0.269	2.418 ± 0.741	2.298 ± 0.569	0.473

The bold P value suggests a significant difference between the variables in the two cohorts.

PCNSL, primary central nervous system lymphoma; aGBM, atypical glioblastoma.

### Efficacy Analysis of Diagnostic Models and Radiologists’ Assessment in Differentiating Primary Central Nervous System Lymphoma From Atypical Glioblastoma

**Table 5** exhibits the diagnostic performance of different models and radiologists’ assessment. For univariate quantitative parameters analyses, compared to models 4 (rT_2_) and 6 (rT_1_), model 5 (T_2_/T_1_) achieved higher efficacy, with an AUC of 0.805 [95% confidence interval (CI), 0.718–0.893] for the training cohort, 0.719 (95% CI, 0.593–0.844) for the test cohort, and 0.822 (95% CI, 0.752–0.892) for the overall cohort, for distinguishing PCNSL from aGBM. For multiple variable combination analysis, models 2 and 3 were constructed with quantitative (rT_2_ + T_2_/T_1_ + rT_1_) and qualitative parameters (localization + incision sign + reef sign + peritumoral leukomalacia sign + T_2_ pseudonecrosis sign), respectively. The diagnostic performance of model 2 is better than that of model 3, with an AUC of 0.826 (95% CI, 0.709–0.885) for the training cohort, 0.778 (95% CI, 0.624–0.877) for the test cohort, and 0.833 (95% CI, 0.754–0.892) for the overall cohort, for distinguishing PCNSL from aGBM. When all the quantitative and qualitative parameters were combined, model 1 achieved the highest diagnostic efficiency, with an AUC of 0.899 (95% CI, 0.828–0.969) for the training cohort, 0.794 (95% CI, 0.666–0.922) for the test cohort, and 0.869 (95% CI, 0.807–0.932) for overall cohort.

**Table 5:** Diagnostic efficacy of different models and radiologists’ assessment in differentiating PCNSL from aGBM.

	Cohort	AUC (95% CI)	ACC (95% CI)	Sensitivity	Specificity	PPV	NPV	*P*
Model 1	Training	0.899 (0.828–0.969)	0.929 (0.858–0.971)	0.813	0.985	0.963	0.916	**<0.001**
	Test	0.794 (0.666–0.922)	0.837 (0.693–0.932)	0.625	0.963	0.909	0.812	**0.002**
	Overall	0.869 (0.807–0.932)	0.901 (0.839–0.945)	0.771	0.968	0.925	0.891	**<0.001**
Model 2	Training	0.826 (0.709–0.885)	0.857 (0.772–0.919)	0.625	0.969	0.909	0.842	**0.016**
	Test	0.778 (0.624–0.877)	0.814 (0.666–0.916)	0.500	1.000	1.000	0.771	**0.006**
	Overall	0.833 (0.754–0.892)	0.872 (0.806–0.923)	0.667	0.979	0.941	0.851	**<0.001**
Model 3	Training	0.797 (0.768–0.884)	0.765 (0.669–0.845)	1.000	0.652	0.582	1.000	**0.031**
	Test	0.750 (0.682–0.873)	0.721 (0.563–0.845)	1.000	0.556	0.571	1.000	0.134
	Overall	0.823 (0.785–0.882)	0.780 (0.703–0.846)	1.000	0.667	0.608	1.000	**0.001**
Model 4	Training	0.751 (0.661–0.841)	0.827 (0.73–0.896)	0.531	0.969	0.895	0.810	**<0.001**
	Test	0.744 (0.609–0.879)	0.791 (0.639–0.899)	0.562	0.926	0.818	0.781	**0.017**
	Overall	0.749 (0.675–0.824)	0.816 (0.742–0.876)	0.542	0.957	0.867	0.802	**<0.001**
Model 5	Training	0.805 (0.718–0.893)	0.857 (0.772–0.919)	0.656	0.955	0.875	0.851	**<0.001**
	Test	0.719 (0.593–0.844)	0.791 (0.639–0.899)	0.438	1.000	1.000	0.750	**0.017**
	Overall	0.822 (0.752–0.892)	0.858 (0.789–0.911)	0.708	0.936	0.850	0.861	**<0.001**
Model 6	Training	0.635 (0.539–0.731)	0.697 (0.539–0.828)	0.188	1.000	1.000	0.675	0.216
	Test	0.594 (0.495–0.693)	0.714 (0.614–0.801)	0.406	0.864	0.591	0.750	0.227
	Overall	0.617 (0.538–0.696)	0.688 (0.604–0.763)	0.396	0.839	0.559	0.729	0.268
Radiologist A	Overall	0.732 (0.579–0.859)	0.724 (0.577–0.852)	0.736	0.710	0.726	0.720	**0.047**
Radiologist B	Overall	0.811 (0.668–0.931)	0.829 (0.783–0.899)	0.857	0.831	0.774	0.896	**<0.001**

The bold P value suggests a significant difference between the variables in the two cohorts. Model 1, rT_2_ + T_2_/T_1_ + rT_1_ + Localization + Incision sign + Reef sign + Peritumoral leukomalacia sign + T2 pseudonecrosis sign; Model 2, rT_2_ + T_2_/T_1_ + rT_1_; Model 3, Localization + Incision sign + Reef sign + Peritumoral leukomalacia sign + T2 pseudonecrosis sign; Model 4, rT_2_; Model 5, T_2_/T_1_; Model 6, rT_1_.

PCNSL, primary central nervous system lymphoma; aGBM, atypical glioblastoma; CI, confidence interval; AUC, area under the curve; ACC, accuracy; PPV, positive predictive value; NPV, negative predictive value.

For radiologist’s assessment, the diagnostic performance of radiologist B with more experience (AUC = 0.811, ACC = 0.829, sensitivity = 0.857, and specificity = 0.831) was better than that of radiologist A (AUC = 0.732, ACC = 0.724, sensitivity = 0.736, and specificity = 0.710).

### Reproducibility of Signal Intensity Ratio Measurement and Radiologist’s Assessment

**Table 6** shows that both inter-reader agreement for SIR measurement and intra-reader agreement for radiologist’s assessment achieved good performance, with ICC/Kappa value ranging from 0.796 to 0.913. For SIR measurements, inter-reader agreement was highest for the measurement of rT_2_ (ICC = 0.913). Regarding reproducibility of radiologist’s assessment, experienced radiologist B (Kappa = 0.903) showed higher intra-reader agreement than that of radiologist A (Kappa = 0.796).

**Table 6:** Reproducibility of signal intensity ratio measurements and radiologists’ assessment.

Variable	ICC/Kappa	95% CI
rT_2_	0.913	0.811–0.952
rT_1_	0.892	0.833–0.945
rT_1_CE	0.876	0.798–0.967
Radiologist A	0.796	0.654–0.869
Radiologist B	0.903	0.832–0.988

ICC, intraclass correlation coefficient; CI, confidence interval.

## Discussion

Differentiating PCNSL from aGBM (with no visible necrosis) is challenging. In the present study, we found that T_2_ pseudonecrosis sign, incision sign, reef sign, and peritumoral leukomalacia sign were closely related to PCNSL. Compared to radiologist’s assessment, model 1, which combined the SIRs and MRI morphological features, achieved the best diagnostic performance in distinguishing PCNSL from aGBM.

During the past decades, various MR modalities and different analysis strategies were explored to differentiate PCNSL from GBM (7–10, 13, 14, 16, 17), whereas the present study focused on SIR analysis of conventional MR sequences mainly based on the following four considerations. First, in clinical practice, T_1_WI, T_2_WI, and T_1_CE imaging are routinely obtained for patients across different hospitals (18). In contrast, advanced MRI techniques, such as diffusion-weighted imaging (DWI) and perfusion-weighted imaging (PWI), are performed when necessary, which require additional expense and time. Furthermore, no unified standard was established for differential diagnosis. For example, although several prior studies have confirmed the efficiency of DWI in distinguishing PCNSL from GBM, overlapping of parameters makes accurate differential diagnosis challenging (19–21). Likewise, PWI is another commonly used technique, and its quantitative measurement reproducibility leads to the lack of a unified threshold to distinguish the two entities (22, 23). Second, radiomics approach can be used for differential diagnosis of PCNSL and GBM. Despite promising results, a recent systematic review suggested that conclusions derived from radiomics should be interpreted with caution due to the suboptimal quality of the studies (17). In contrast, the traditional analysis method is time-saving and easy for clinical implementation and interpretation. Third, clinical experience of radiologists suggests that PCNSL has slightly higher T_1_WI and lower T_2_WI signal intensity than GBM. However, visual judgment is subjective, and precise quantitative assessment is needed, especially for those that cannot be differentiated by the naked eye. Although T_1_ and T_2_ mapping can accurately quantify T_1_ and T_2_ values of tissue, they are not performed as routine sequences due to long scanning time and complex postprocessing. In contrast, signal intensity of the lesion is easily obtained from T_1_WI and T_2_WI but is susceptible to many factors, including the characteristics of the tissue itself (T_1_ value, T_2_ value, and proton density) and MRI equipment and scanning parameters (field strength, repetition time, and echo time). Therefore, in this study, the SIR was used as a quantitative parameter to eliminate the influence of different MRI scanners and imaging parameters on the results. Similar to our study design, the SIR also showed potential for differential diagnosis in other scenarios (12, 24–26). However, different from previous studies, we used an external test cohort to further clarify the actual diagnostic performance of the SIR. Fourth, our study did not involve complex image preprocessing, including image registration, brain extraction, and standardization. ITK-SNAP software used in our study can realize simultaneously quantitative measurement of T_1_WI and T_2_WI signal intensity in the same ROI without image registration. The entire analysis was limited to the time required to identify lesions and electronically locate ROIs. From the clinical point of view, this approach may be a highly cost-effective quantitative analysis tool.

Most previous studies enrolled all PCNSL and GBM cases, regardless of sign of intratumoral necrosis as a powerful indicator to distinguish the two entities, and their inclusion criteria could partially explain the higher ACC (7, 8, 10, 27). Therefore, we reasoned that confining our study to PCNSL and aGBM cases is closer to the clinical diagnostic dilemma in order to seek more powerful imaging signs to identify the two entities. In our study, four morphological features were closely associated with PCNSL, including incision sign, T_2_ pseudonecrosis sign, reef sign, and peritumoral leukomalacia sign. Among them, the diagnostic value of incision sign has been confirmed in a previous study (28). T_2_ pseudonecrosis sign, reef sign, and peritumoral leukomalacia sign, defined by the present study for the first time, were observed only in PCNSL and not in aGBM. For T_2_ pseudonecrosis sign, the mismatch between heterogeneous T_2_WI signals and homogeneous enhancement is the diagnostic core, which may be related to the degree of tumor infiltration along the white matter fiber bundles. The reef sign was defined as single or multiple foci that presented as hypointensity on T_1_WI, hyperintensity on T_2_WI, and brighter signal within contrast-enhanced area of the lesion. Although the corresponding pathological mechanism of this sign is still unclear, it may be related to the leakage of contrast medium in the tumor area (29). Peritumoral leukomalacia sign was defined as an area manifested as hypointensity on T_1_WI and hyperintensity on T_2_WI in the region adjacent to the tumor. The possible explanation is that the PCNSL cells are closely arranged and cluster along vascular channels, which destroy the blood supply of the adjacent brain parenchyma, resulting in encephalomalacia (30). The above four imaging signs were statistically significant between PCNSL and aGBM based on the overall cohort. So, we believe that these signs may be useful in daily radiological practice and help differentiate PCNSL and aGBM.

In the present study, PCNSL had higher rT_1_ and lower rT_2_ than aGBM. The possible mechanism is that a high degree of cellularity and high nuclear–cytoplasm ratio lead to the decrease of tumor water content (31, 32), which contributes to signal characteristics. Although the rT_1_CE of PCNSL was slightly higher than that of GBM, there was no significant difference between the two groups. This result differs from that of the study by Anwar et al. (9), which reported a sensitivity of 83.3%, specificity of 85.7%, and AUC of 0.92 for differentiating PCNSL and GBM. Different study populations, MRI sequence parameters, and timing and dosage of MRI contrast administration may contribute to this inconsistency. Notably, compared with rT_2_ or rT_1_, T_2_/T_1_ achieved the highest AUC in distinguishing PCNSL from aGBM. The good diagnostic performance may be attributed to the fact that T_2_/T_1_ can provide better contrast as a quantitative tool. Many studies have confirmed that T_1_/T_2_ ratio was useful in differentiating benign and malignant lesion in breast (33) and liver (24), quantifying the demyelinated cortex in multiple sclerosis (25).

There are several limitations for the current study. First, our sample size was relatively small, especially for the aGBM group. From 3 medical centers, only 48 patients with atypical and solid enhancement met the inclusion criteria and were selected. Second, radiologic–pathologic correlation for morphological features was not performed. Third, although the repeatability and reproducibility of SIR measurements were good; however, possible bias still existed due to the manual positioning ROIs. Finally, our cohort included heterogeneous MRI equipment and scanning parameters mimicking the circumstances encountered in a clinical setting. However, as a semiquantitative parameter, how SIR is affected by different equipment and scanning parameters is not clear. Further prospective study is needed.

## Conclusion

T_2_ pseudonecrosis sign, reef sign, and peritumoral leukomalacia sign are closely related to PCNSL, which are never reported before. Compared to radiologists’ assessment, the combination model of morphological features and SIRs can provide better diagnostic performance in distinguishing PCNSL from aGBM.

## Data Availability Statement

The data analyzed in this study are subject to the following licenses/restrictions: The raw data are not publicly available due to them containing information that could compromise research participant privacy/consent. Requests to access these datasets should be directed to hanyu0920@163.com.

## Ethics Statement

The studies involving human participants were reviewed and approved by the institutional review board from Tangdu Hospital, XD Group Hospital, and West China Hospital. Written informed consent for participation was not required for this study in accordance with the national legislation and the institutional requirements.

## Author Contributions

G-BC and L-FY conceived the study. YH, Z-JW, and W-HL participated in the study design. YH, Z-JW, W-HL, YY, JZ, X-BY, LZ, GX, S-ZW, and L-FY performed the data acquisition. L-FY and YH participated in the statistical analyses. All authors participated in the data interpretation. YH drafted the first version of the report. All authors contributed to the article and approved the submitted version.

## Funding

This study received financial support from the Key Industrial Chain Projects in the Field of Social Development of Shaanxi Province (No. 2019ZDLSF02-07 to G-BC), National Natural Science Foundation of China (No. 82102127 to YY) and the General Projects in the Field of Social Development of Shaanxi Province (No. 2019SF-002 to L-FY).

## Conflict of Interest

The authors declare that the research was conducted in the absence of any commercial or financial relationships that could be construed as a potential conflict of interest.

## Publisher’s Note

All claims expressed in this article are solely those of the authors and do not necessarily represent those of their affiliated organizations, or those of the publisher, the editors and the reviewers. Any product that may be evaluated in this article, or claim that may be made by its manufacturer, is not guaranteed or endorsed by the publisher.
