# Supplementary material for: Differentiation Between Primary Central Nervous System Lymphoma and Atypical Glioblastoma Based on MRI Morphological Feature and Signal Intensity Ratio: A Retrospective Multicenter Study
Source: Front Oncol. 2022 Jan 31;12:811197. doi: 10.3389/fonc.2022.811197 (PMC8841723; doi:10.3389/fonc.2022.811197)
Supplement: Supplementary file 1 [file DataSheet_1.docx]

**Table S1** Detailed information of MRI parameters.

|  | **Tangdu Hospital** | | |  | **XD Group Hospital** | | |  | **West China Hospital** | | |
| --- | --- | --- | --- | --- | --- | --- | --- | --- | --- | --- | --- |
|  | **T_1_WI** | **T_2_WI** | **T_1_CE** |  | **T_1_WI** | **T_2_WI** | **T_1_CE** |  | **T_1_WI** | **T_2_WI** | **T_1_CE** |
| **Manufacturer** | GE 3.0 T  SIEMENS 1.5T | | |  | Philips 1.5 T | | |  | GE 1.5 T  SIEMENS 3.0 T  Philips 1.5 T | | |
| **TR ( ms)** | 450-1750 | 4247-4300 | 450-1750 |  | 476 | 3014 | 542 |  | 300-500 | 2500-5000 | 300-500 |
| **TE ( ms)** | 10-24 | 93-109 | 8.9-24 |  | 15 | 80 | 17 |  | 8-12 | 84-104 | 8-12 |
| **Slice thickness ( mm)** | 5-5.5 | 5-5.5 | 5-5.5 |  | 6 | 6 | 6 |  | 5.5-6 | 5.5-6 | 5.5-6 |
| **Slice spacing ( mm)** | 1-1.5 | 1-1.5 | 1-1.5 |  | 1.5 | 1.5 | 1.5 |  | 1-1.5 | 1-1.5 | 1-1.5 |

Note: T_1_WI = T_1_-weighted image; T_2_WI = T_2_-weighted image; T_1_CE = contrast-enhanced T_1_-weighted image; TR = repetition time; TE = echo time; In Tangdu hospital, axial T_1_CE were obtained 3 min 20 s after intravenous bolus injection of 0.1 mmol/kg bodyweight of gadodiamide (Omniscan; GE Healthcare, Co. Cork, Ireland); In XD Group hospital, axial T_1_CE were obtained 3 min 11 s after intravenous bolus injection of 0.1 mmol/kg bodyweight of gadodiamide (Gadopentetate Meglumine; Guangzhou Consun Pharmaceutical Co. Ltd); In West China hospital, axial T_1_CE were obtained 3～5min after intravenous bolus injection of 0.1 mmol/kg bodyweight of gadodiamide (Omniscan; GE Healthcare, Co. Cork, Ireland).


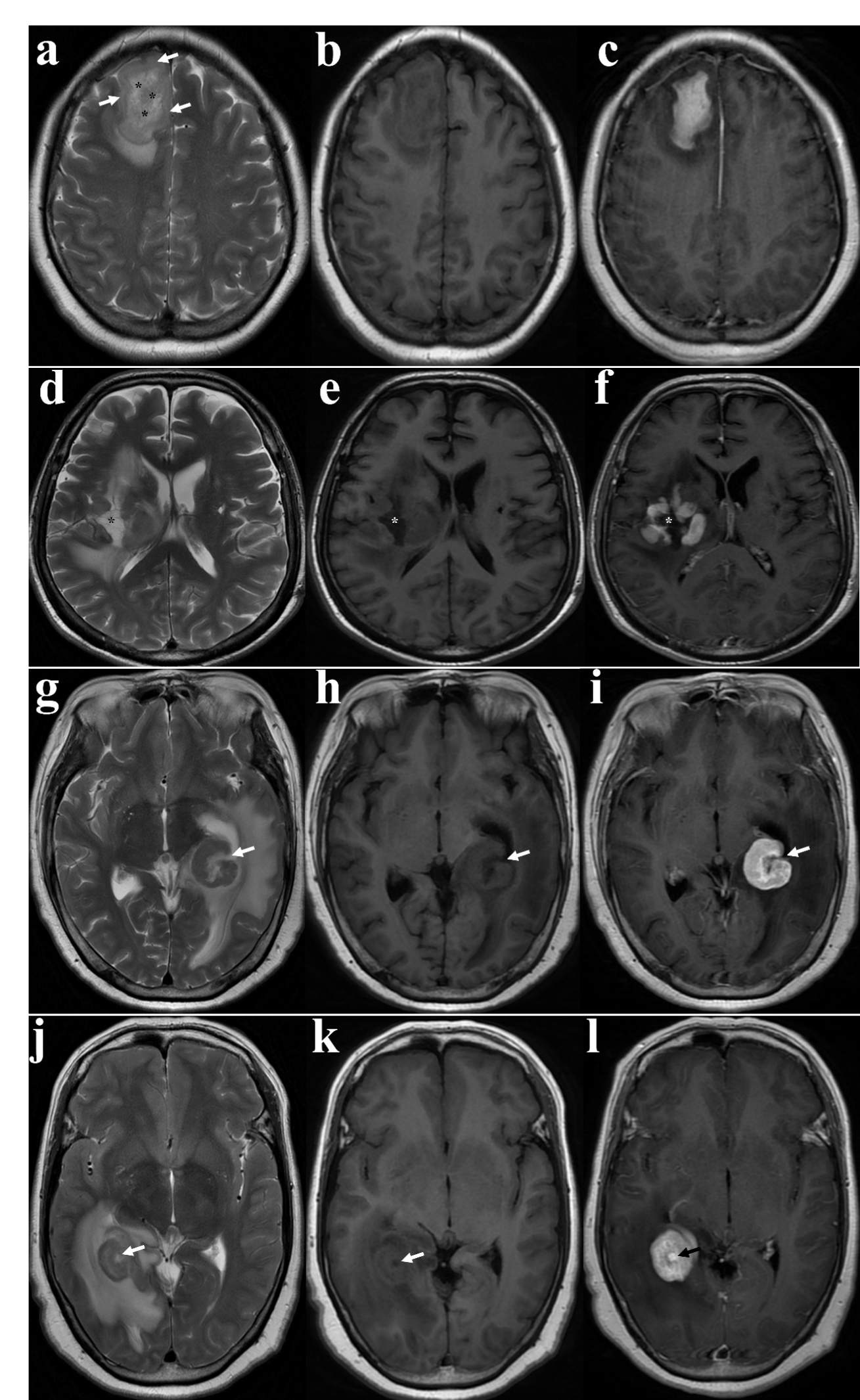


**Fig. S1.** Representative cases of imaging signs

**a-c**, T_2_ pseudonecrosis sign: diagnostic core of this sign is the mismatch between heterogeneity on T_2_ weighted imaging (T_2_WI) and homogeneous enhancement on contrast enhanced T_1_ weighted imaging (T_1_CE). On T2WI (a), the edge of the tumor (white arrow) is isointense to slightly hyperintensity (gray matter as reference), and the inhomogeneous hyperintensity inside the tumor (black asterisk) is similar to tumor necrosis. However, after the injection of contrast agent, the entire tumor shows uniform enhancement on T_1_CE(c). So it's called T_2_ pseudonecrosis sign.

**d-f**, peritumoral leukomalacia sign: diagnostic core of this sign is the coexistence of tumor and adjacent leukomalacia. Peritumoral leukomalacia refers to the area (asterisk) adjacent to the tumor shows hypointensity on T_1_WI (e), hyperintesity on T_2_WI (d) and no contrast-enhancement on T_1_CE(f).

**g-i**, incision sign: diagnosis of this sign is mainly based on T_1_CE images (axial, sagittal or coronal plane). This sign appears as 1-2 umbilical concave or striated defects on the edge of the enhanced lesions. In this case, an umbilical concave (white arrow) can be seen at the edge of the enhanced lesion on T_1_CE (i), which presents as hypointensity on T_1_WI (h), hyperintesity on T_2_WI (g).

**j-l**, reef sign: there is a reef-like foci within the lesion, presenting as hypointensity (white arrow) on T_1_WI (k), hyperintensity (white arrow) on T_2_WI (j) and brighter signal (black arrow) in the context of enhanced lesion on T_1_CE (l).


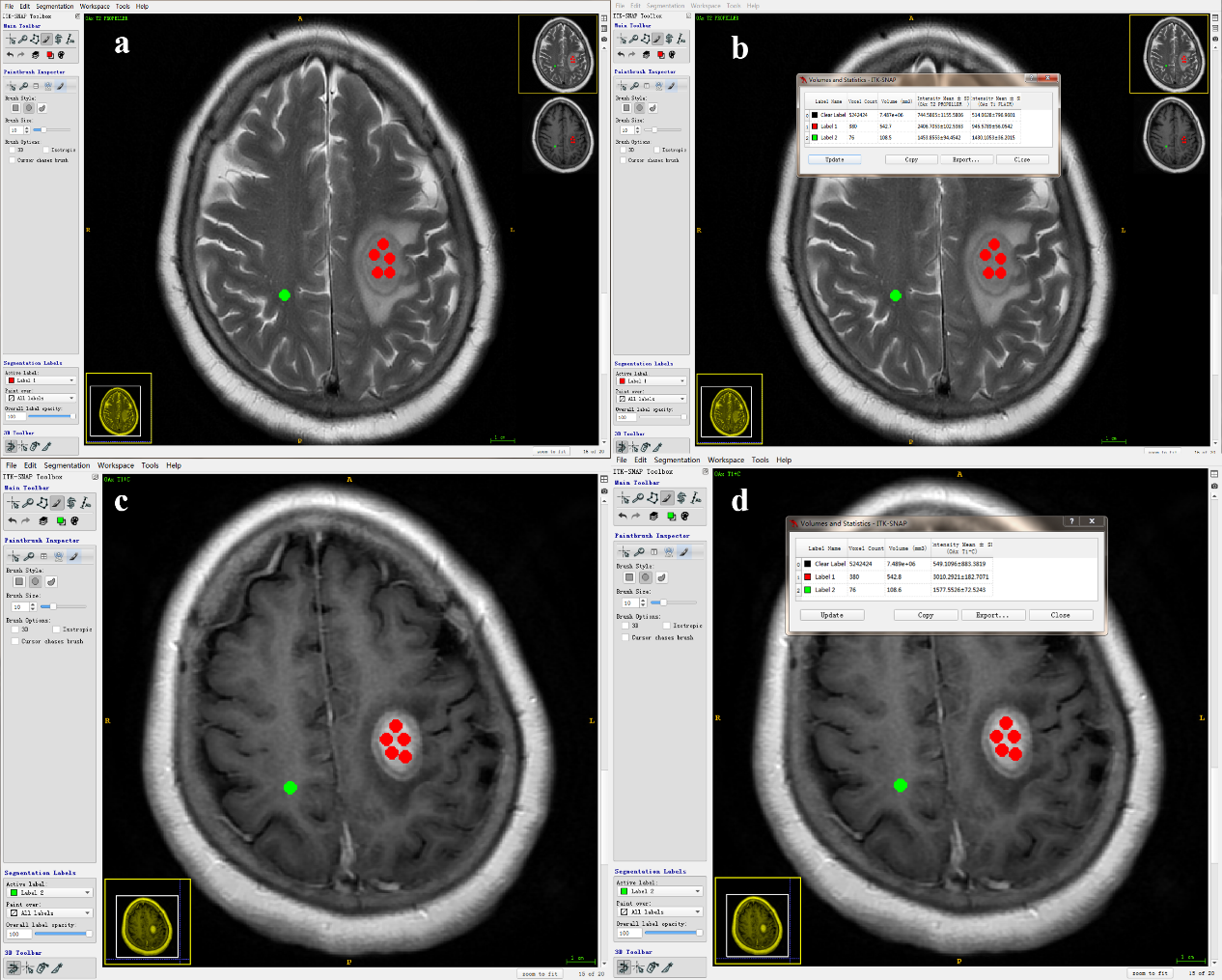


**Fig. S2.** Details of ROI placement strategy

**a.** T_2_WI and T_1_WI in DICOM format are loaded into ITK-SNAP software. In brush mode, round brush is selected and the brush size is 10 (voxel count = 76; area = 21.7 mm^2^). The radiologists carefully reviewed all the layers of the tumor. Five red circular ROIs (Label 1) are placed in the relatively lower signal area of the lesion on T_2_WI (it should be noted that the five ROIs are not required to be on the same cross-section). The same ROIs are automatically placed in the corresponding position of T_1_WI. To perform the normalization, a green ROI of the same size (Label 2) is placed in contralateral normal white matter. Care is taken to avoid high signal area of white matter. **b.** Using the statistical function of the software, the quantitative mean signal intensity value of ROI is recorded.

**c-d**. the same method is used to measure T_1_CE ratio. Different from the former analysis, ROI is placed in the area with relatively higher signal area of the lesion on T_1_CE by adjusting the image contrast.

**Note:** In our study, the size of ROI was not randomly designed. In order to ensure the repeatability of SIR measurement, we evaluated the impact of different ROI sizes on the repeatability of SIRs before the start of the study. The detailed process is as follows: MRI data from 40 patients were randomly selected. ROI placement was done by a neuroradiologist (X.G., 10 years of experience in brain tumor diagnosis), according to the principles described above (**Fig. S2**). For each patient, we measured four times according to different ROI sizes (brush sizes are set to 5, 10, 15 and 20 respectively). Then, the quantitative parameters (rT_2_, rT_1_ and rT_1_CE) corresponding to each ROI size were recorded. To assess intra-observer agreement, the same radiologist re-evaluated the MR images after a one months’washout period. Finally, the ROI size used for the study was determined based on the intra-observer consistency of the quantitative parameters (**Table S2**). Therefore, we chose a brush size of 10 as the size of ROI.

**Table S2** Repeatability of signal intensity ratio measurement for different brush size.

| **Brush size** | **ICC_rT2_ (95%CI)** | **ICC_rT1_ (95%CI)** | **ICC_rT1CE_ (95%CI)** |
| --- | --- | --- | --- |
| **5** | 0.782 (0.687-0.895) | 0.778 (0.663-0.887) | 0.812 (0.713-0.895) |
| **10** | 0.921 (0.845-0.986) | 0.901 (0.878-0.977) | 0.877 (0.786-0.949) |
| **15** | 0.836 (0.746-0.944) | 0.876 (0.708-0.939) | 0.867 (0.801-0.935) |
| **20** | 0.864 (0.809-0.953) | 0.829 (0.795-0.901) | 0.788 (0.661-0.883) |

Note: ICC = intraclass correlation coefficient; CI = confidence interval
